# Chasing Waterfalls: A Cascade Mechanism to Generate Triplets from ^2^LMCT States

**DOI:** 10.1021/acscentsci.5c01666

**Published:** 2025-09-20

**Authors:** Alexandra T. Barth, Felix N. Castellano

**Affiliations:** Department of Chemistry, 6798 North Carolina State University, Raleigh, North Carolina 27695-8204, United States

## Abstract

Earth-abundant ligand-to-metal charge transfer (LMCT) chromophores in donor–acceptor dyads unlock an electron transfer pathway for efficient triplet state formation.

## Graphical abstract

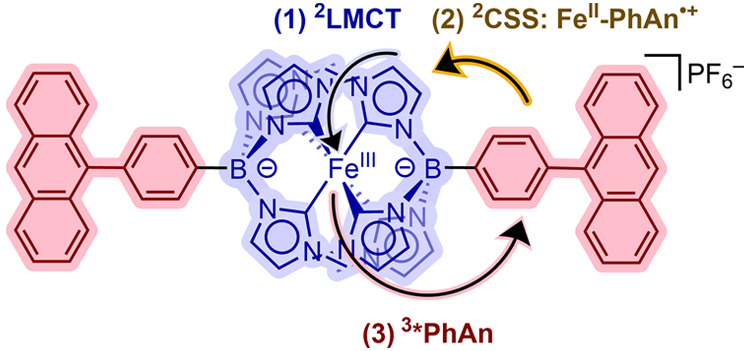

Light-activated molecules that generate value-added materials offer a bright alternative to nonrenewable fuels in tackling the sustainable energy crisis. However, one central challenge is identifying earth-abundant materials that can efficiently harvest solar light for difficult photochemical transformations. To address this problem, in this issue of *ACS Central Science*, Felix Glaser, Alejandro Cadranel, Ludovic Troian-Gautier, and co-workers offer a new strategy to utilize the short excited-state lifetimes of Fe^III^ complexes in donor–acceptor systems.^1^ While investigating the excited state properties of Fe^III^ metal–chromophore dyads, their work reveals that chromophore triplet formation occurs through an electron transfer intermediate. This enables the intermediate state energy to be tuned through solvent selection, providing environmental control over intramolecular charge transfer efficiency and dynamics.

Long-lived photosensitizers often rely on noble metals such as Ir^III^ and Ru^II^ to efficiently harvest solar light. Synthetic approaches have broadened the availability of chromophores for photochemistry, especially by utilizing ligand-to-metal charge transfer (LMCT) activation of earth-abundant early transition metals and open-shell species.^2,3^ LMCT chromophores represent a growing frontier in earth-abundant photocatalysis. Among the few known examples, Fe^III^ *N*-heterocyclic carbenes exhibit desirable properties for photochemistry, including emissive ^2^LMCT excited states.^4^ This chromophore class has already been shown to mediate electron transfer reactions using green light.^5^

The authors overcome the photophysical limitations of LMCT chromophores by developing a molecular dyad that pairs an Fe^III^ photosensitizer with an anthracene quencher. Their previous report on this molecule revealed that this dyad has improved photophysical properties when compared to the bare Fe^III^ photosensitizer, generating an 11.5 μs anthracene-localized triplet lifetime and an order-of-magnitude increase in cage escape yields.^6^ However, their prior study assigned that a doublet-triplet energy transfer (DTET) mechanism mediates this charge reorganization.

Here, this assignment was further probed using ultrafast transient absorption spectroscopy. Instead of observing direct energy transfer from the initial *Fe^III^ state, a multistep process was revealed. After light absorption (^2^LMCT, ∼2.15 eV), ultrafast intramolecular electron transfer generates a new short-lived excited state intermediate, assigned as a charge-separated state (^2^CSS, ∼1.9–2.1 eV). This intermediate, which forms within nanoseconds, then rapidly converts into the persistent, triplet state localized onto the 9-phenylanthracene (^3^*PhAn, ∼1.8 eV) subunit, as shown in Figure 1. This is akin to experimental results in biological photosynthetic systems^7,8^ and synthetic analogues of these systems,^9,10^ which implicate photoinduced charge recombination in generating triplet states. The final ^3^*PhAn triplet state is nonemissive, long-lived, and chemically reactive.^6^ While the absorption and photoluminescence emission spectra are unaffected by solvent choice, the solvent polarity influences the ^3^*PhAn formation quantum yield and ^2^CSS lifetime, implying that the ^2^CSS energy is indeed sensitive to solvent changes.

**Figure 1:**
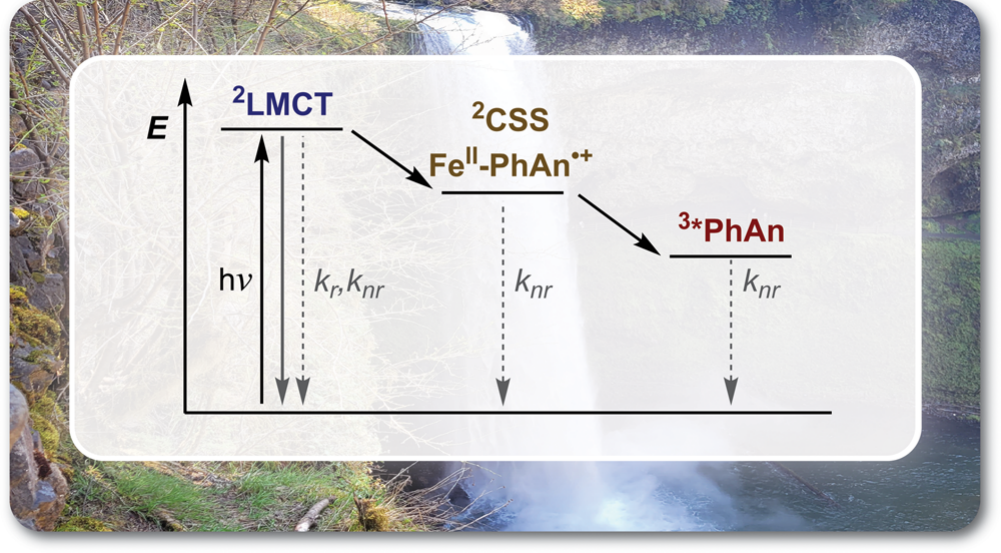
A simplified energy level scheme detailing the excited state deactivation cascade of [Fe­(L^PhAn^)_2_]^+^. LMCT = ligand-to-metal charge transfer. CSS = charge separated state. PhAn = 9-phenylanthracene. Radiative transitions are shown as solid lines. Nonradiative transitions are shown as dashed lines. Reproduced from ref 1. Available under a CC-BY 4.0 license. Copyright 2025 Felix Glaser, Giovanni M. Beneventi, Alejandro Cadranel, and Ludovic Troian-Gautier. Photograph included with permission from the copyright holder.

By revealing a hidden, multistep electron transfer process, this work offers a rational strategy to overcome some of the primary limitations of earth-abundant photosensitizers.

In noble metal d^6^ metal–chromophore dyads, it is typical for energy transfer to be mediated by the long-lived ^3^MLCT state, which is accessed through intersystem crossing within the MLCT manifold. For 3d metal complexes, the inaccessibility of intersystem crossing being competitive with other excited state decay processes represents a significant barrier to maintaining long-lived excited states. However, within this work, the LMCT chromophore dyad utilizes the short-lived (∼ns) excited states of the parent complex to mediate efficient electron transfer, overcoming both intermediate energy-loss pathways and diffusional limitations. This mechanism enables large quantum yields of triplet formation, ranging from 5% in acetonitrile to 75% in dichloromethane, dependent on the relative polarity of the solvent.

This mechanism offers a new approach to generating triplet states from LMCT chromophores rather than aiming to directly reproduce the photochemistry of its second- and third-row transition metal analogues.

Instead of trying to extend the lifetimes of Fe^III^ chromophores to drive excited-state electron transfer, the current strategy enables rapid and efficient unimolecular triplet-state generation using energetically matched chromophores. While these efficiency gains hinge on direct covalent attachment, as demonstrated in this work, this result is still a major step forward toward designing tunable photosensitizers from iron, Earth’s most abundant transition metal element.
